# 

**Published:** 1919-04-05

**Authors:** 


### THE HOSPITAL

*THE MODERN NEWSPAPER OF ADMINISTRATIVE MEDICINE AND INSTITUTIONAL LIFE*

INCLUDING

ADMINISTRATION, NATIONAL INSURANCE, HEALTH

AND

THE INTERESTS OF ALL INSTITUTIONAL WORKERS

APRIL TO SEPTEMBER 1919

VOLUME LXVI

SURGEON GENERAL'S 241408 LIBRARY

LONDON

PUBLISHED BY THE SCIENTIFIC PRESS (LIMITED), AT *THE HOSPITAL* BUILDING

28 & 29 SOUTHAMPTON STREET, STRAND, W.C. 2.

MDCCCCXIX

W1
HO69X

### INDEX TO VOL. LXVI.

Adami, Col. J. G., 235
Advertisement, A "Physician's," 236, 282
Aerial Nurses, 296
Afghan War and Khyber Pass, The, 289, 317, 445, 467, 493, 511, 521, 531, 539, 561
African Bibi Nursing Corps, 250
Agnew, Mr. C. Morland, 282
Agricultural Costings Committee, 468
Air Force Hospitals, 23, 156; Medical Service, 502
Albuminuria, 425
Alcohol, The Question of, 399
Allbutt, Sir T. Clifford, Presentation Portrait, 279
Altrincham, "John Leigh" Memorial Hospital, 170
Ambulance Trains, 330
Ambulance Provision, 38
America (*see* United States)
American Distinguished Service Medal, 330
American Hospital at Highgate, 54; in London, 143, 401
Anæsthesia, 205
Anatomical Committee of London, 316
Animals, Experiments on, 533
Annual Meetings, 22, 118, 163, 194, 300
Annuities of Nurses, 114
Ante-Natal and Intra-Natal Care, 420
**APPOINTMENTS: MATRONS' AND SISTERS'**, 50, 74, April 26, vi, May 3, v June 28, iv, 369, 398, July 19, viii, 464, 486, August 16, viii, August 23, iv, August 30, viii, 600, 622; Medical, 256, 367, August 23, iv
[illegible]hangel, Wounded from, 283
[illegible].c, Hospitals in the, 166
[illegible]ny Case Sheets, The Production of, 237
[illegible]rmy Chaplain, The, 95
[illegible]rmy Council and V.A.D.s, 112
Army, Doctors and the, 583
Army Medical Stores, Surplus, 236
Army Nurses, 44; War Memorial, 340; Pay, 397; Disabled Nurses, 434; Appreciation of, 559; Out of Work, 526; New Orders, 562; Pensions, 596; Unemployed Pay, 596
Artificial Appliances for Officers and Men, 477
Artificial Limbs, A Demonstration of, 472
Ashdown, Miss, 249
Association of Hospital Matrons, 112, 155, 161, 295, 356
Asthma, The Treatment of, 497
Asylum Workers' Association, 249
Asylum Workers' Charter, Supp. June 28, July 28
Australian Notes, 96
Australian Nurses, 296
Australian Trained Nurses' Association, 547
Author to read in Hospital, The, 204
Auxiliary Hospitals' Surplus Stocks, 123
Awakening of the Hospitals, The, 75

Baby Farmer Sentenced, 640
Baby Week (*see* Infant Welfare)
Bacon, Miss A., 460
Bacteriology, A Chair of, 235
Barling, Col. G., 3
Barwell, Miss M., 192
Bateman, Dr. R. G., 78
Battersea Borough Council, 259
Bax, Capt. R. E. V., 4
Becher, Dame E. A., 353
Bedfordshire Hospital Guild, 114
Bed Sores, Formalin Treatment of, 520
Bee-keeping, Institutional, 204; Stings, 483, 583
Bermondsey Board of Guardians, 204, 225, 373, 482
Bermondsey Borough Council, 318, 490
Bermondsey Infirmary, 53, 192, 226, 528
Berwick, Mrs. M., 250
Bethnal Green Nurseries, 458
Betrayal and Compromise, 89
Bilharziasis and its History, 375
Birmingham: Hospital Council, 79; Professions and Trades Fund, 80; Co-ordination of Hospitals, 261; Board of Guardians, 342; Voluntaryism, 448; District Nursing Society, 460; Dudley Road Poor-Law Infirmary, 482; Mr. Julius Jones's Legacies, 536; Accident to Nursing Sisters, 618
Birth Rate, 142
Blind, The: The New Treatment, 407; State Assistance, 416, 527; Books, 599
Blood Transfusion, 449
Board of Education, 115, 434; Grants to Ex-Service Men, 513; Fruit Canning, 546
**BOOK WORLD:**
Balfour: Dr. Elsie Inglis, 108
Chance: Bodily Deformities, 244
Chandhuri: Sir William Ramsay as a Scientist and Man, 107
Clayton-Greene: Pye's Surgical Handicraft, 244

BOOK WORLD—*continued*.
Corbett-Smith: The Problems of Sex Disease, 567
Dentists' Register for 1919, 567
Dowd: The Doings of Donovan in and out of Hospital, 108
Dumas and Carrel: Technique of the Irrigation Treatment of Wounds, 454
Flexner: Prostitution in Europe, 345
Fox: Dr. John Fothergill and his Friends, 345
Gallichan: A Text-Book of Sex Education, 346
Goodall-Copestake: Massage as a Career for Women, 244; Theory and Practice of Massage, 453
Gordon: Rhymes of the Red Triangle, 567
Gould: Elements of Surgical Diagnosis, 244
Halliburton: Handbook of Physiology, 244
Hurry: Vicious Circles in Disease, 453
Hutchinson: The Doctor in War, 243
Lelean: Sanitation in War, 13
Lepine: Mental Disorders of the War, 108
Lewis's Medical and Scientific Circulating Library, Catalogue of, 454
Luff: A Manual of Chemistry, 13
McCullum: The Newer Knowledge of Nutrition, 453
MacCurdy: War Neuroses, 107
MacKenna: The Adventure of Life, 108
MacKenzie: The Future of Medicine, 466, 622
Makins: On Gunshot Injuries to the Blood Vessels, 453
Medical Register for 1919, 567
Military Medical Manners, 108
Mitchell: Bydand: Poems of War and Peace, 453
Moore: The History of St. Bartholomew's Hospital, 11
Morgan: Eugenics and Environment, 567
Muir, R.: Manual of Bacteriology, 244
Muir, E.: Kala-Azar: its Diagnosis and Treatment, 244
Otabe: The Science and Art of Deep Breathing, 13
Page: A Medical Field Service Handbook, 346
Prenderville: Anæsthetics and the Nurses' Duties, 454
Pritchard: The Physiological Feeding of Children, 454
Richards: The Cost of Food, 244
Rogers: Fevers in the Tropics, 244

BOOK WORLD—*continued.*
Savage: Food and the Public Health, 567
Vischer: Barbed-wire Disease, 454
Worcester-Drought: Cerebro-Spinal Fever, 346
Zimmern: Electro-Diagnosis in War, 108
Books and the War, 451
Bootman, Miss Mabel, 391
Bottles, Shortage of Medicine, May 31, iv
Bowlby, Sir A., 316
Brighton Poor-Law Infirmary, 354
Bristol's Amalgamation Scheme, 534
British Hospitals' Association, 195
British Medical Association, Special Meeting, 51; Clifford Allbutt Presentation, 279; Panel Doctors, 456
British Science Guild, 382
British Women's Hospital (*see* College of Nursing—Nation's Fund)
Brooks, Sir David, 261
Bruce-Vaughan, Colonel, Memorial to, 605
Brussels, British Farmers' Hospital, 92 160
Bubonic Plague at Liverpool, 201, 208, 467; at Avonmouth, 445; Plague and I.M.S., 496
Buchanan, Lady, 638
Budapesth Hospitals, Nursing Arrangements, 502, 584
Buildings Contemplated and in Progress, 300, 462, 483, 500
**BUREAU OF INFORMATION, OUR** (*see* The Institutional Worker—the Supplement to THE HOSPITAL)
Burnett, Sir E. Napier, 195, 235, 627
Burnley Board of Guardians, 504
Burns, Modern Treatment of, 174
Burrows, Col. H., 99
Bushey Park Children's Sanatorium, 342

"Caduceus," 291
"Ca'ing Canny" Throughout the Nation? 581
Californian Hospitals, 158
Camberwell Borough Council, 260
Cambridge: New Nursing Hostel, 282; Research at, 513
Canadian Nurses: Cost through the War, 192; Eight-Hour Day, 546
Cancer of the Uterus, Inoperable, 105; Rays and the Problem, 127; Imperial Research Fund, 446
Cannes Conference, Tuberculosis, 77, 92
Canterbury, The Archbishop of, 288
Canterbury Maternity Association, 67
Cantlie, Sir James, 315
Cape Hospital Board, 192
Cardiff: Nursing Association, 92; Preventive Medicine at, 170, 202; Prince of Wales's Hospital, 261; Proposed Psychiatric Clinic, 403
Carnarvon Board of Guardians, 504
Cavell, Edith: Home at London Hospital, 67; Account of Last Hours, 68; Funeral, 92, 112, 132, 156, 167, 224; Melbourne Fund, 114; Special Supplement, 177, 210; The Real Test of the Nursing Spirit, 223; Home-coming Sisters, 272; Royal Academy Portrait, 294, 317, 353; A Memento, 390; Northern Counties' Memorial Fund, 460; German Version of Trial, 546; Trial of Quien, 598
Central Bureau Loan Fund, 391
Central Council for District Nursing, 67
Central Midwives Board, 18, 482, 505; Scotland, 157, 639
Cerebro-Spinal Fever, 32
Charity Commissioners, Annual Report of, 101
Charity Voting Reform Association, 374
Charterhouse Military Hospital, 341
Chatham, Royal Naval Hospital, 204
Chelsea, Pimlico, and Belgravia Nursing Association, 226
Chepstow Hospital, 55
Cheshire County Nursing Association, 528
Chester, V.A.D. Service, 415
Child-Bearing, The Mortality of, 265
Child Employment, 402
Children's Country Hospital Fund, 373, 527
Cholera, The Prevention of, 128
Cholera and Sickness, 493
Church, Practitioners and the Marriage of Syphilitics, The, 313
Cinemas: In Mental Hospitals, 56; Teaching Surgery, 373
Civil Re-establishment of Soldiers, 481
Clergyman and Dying Patient, 585
Clergy, Medical Examination for the, 489
Cliveden Hospital, The Migration of, 238
Clonmel Asylum, 330
Coal: Decreased Output Explained, 403; Increased Price, 416
Cod-liver Oil Preparations, 102
**COLLEGE OF NURSING** (*see also* State Registration of Nurses): 1, 15, 42, 44, 49, 74, 89, 90, 111, May 3, vi, 131, May 10, viii, 164, 190, 224, 229, 231, 249, 256, 271, 273, 278, 292, 297, 326, 327, 328, 332, 352, 357, 360, 412, 480, 502, 562, 595, 616, 617; Appointment Inquiry Bureau, 17; The Nation's Fund, 18, 43, 90, 91, 132, 225, 295, 330, 480, 617; Salaries' Committee, Report of, 248, 253, 271, 276, 335, 353; Irish Board, 231; Scottish Board, 231, 486; Nursing Studentships, 434, 442; Sister-Tutor Studentships, 464, 481; Coat of Arms and Badge, 482, 612. Centres: Aberdeen, 398; Birmingham, 256, 414, July 19, viii, 486; Brighton, 164, June 28, iv, 360, August 16, viii, 636; Bristol, 296; Cambridge, 26; Derby, 256, June 28, iv; Edinburgh, May 10, viii, 486; Glasgow, 26; Halifax, Aug. 30, viii; Leeds, 26, Aug. 16, viii; Lincoln, 164, 256; Liverpool, 74, May 24, iv, 360, 398, 622; London, May 3, vi, 210, 256, 278, 300, June 28, iv, 360, 398, 442; Manchester, 26, 295, 297, 442; Newcastle-on-Tyne, 50; Northumberland and Durham, 256; Norwich, 360; Nottingham, 300, 622; Plymouth, Aug. 16, viii; Sheffield, 50, 636.
Collier, The Health of the, 129
Collins, Dr. Frank, 423
Comforts for Sick, Record of, 390
Consumption (*see* Tuberculosis)
Contaminated Foods, 286
Controversy, 111
Convalescent Homes Grouped, 340
Co-operative Wholesale Society, Ltd., 483
Cottage Hospitals after the War, 169
Cottle, Dr. W., 237
Crookes, Sir Wm., 29
Cruelty to Animals, 626
Curry, Mr. W. J., 315, 320, 403

Darlington, The Dilemma at, 203
Davis, A. W., 100, 260
Decubitus, 530
Demobilisation: Military Hospitals, 64; V.A.D. Nurses, 68; Nurses, May 10, viii, 157, 502; Ministry of Labour and Nurses, 225; Y.M.C.A., 324
Dental: Public Dentists, 30; Future of Teaching, 31; Unqualified Dentists, 106; Unregistered Practice, 121; Pharmacists and Dental Register, 262; Hospitals and Dental Service, 323; Isle of Man Register, 477; Dr. King Brown, 490; Treatment, 514; Dentistry as a Profession, 560
Derbyshire County Nursing Association, 113
Derbyshire Nursing and Sanitary Association, 460
Dermatology, The New, 147, 239, 377, 519
Devastated Areas, 597
Devonshire County Nursing Association, 274
Diabetes, Treatment of, 173
Diagnosis, The Art of, 465
Diarrhœa, Epidemic, The Hospital Treatment of, 382
Dick, Mr. L. H. M., 328
Discharged Soldiers: Treatment of, 371; Friends' Fares to Hospital, 397; Neurasthenic Cases, 506; Pensions, 603
Disease and Accident, 429
Disease and the Laboratory, 425, 471, 517
Dispensaries, The Permanence of, 55
Dispensing: Voluntary Hospital Dispensers, 31; Dispensers at Institutions, 146; Register, May 31, iv; Pharmacists and Dental Register, 262; Honour for Hospital Pharmacist, 470; Apothecaries' Assistants and Pharmacists, 506; New Regulations for Pharmaceutical Qualifications, 583
District Nurses: Salaries, 91, 93; Stratford, 134, 228; Directory, 250
Doctors' War Services, Recognition of, 64
Dollis Hill House Hospital, 67
Domestic Service, 68
Domestic Training in Hospital, Supp. May 17, May 24, June 7
Donations Not Recoverable, 32
Dorothy Bags for Soldiers, 547
Drink, Control of the Sale of, 165
Drugs (*see also* Dispensing), The Week's Market In, 6, 32, 102, 124, 146, 170, 204, 238, 262, 284, 318, 302, 404, 424, 448, 492, 516, 586, 602, 628
Dublin, Nurses' Club, 614
Dufferin Association, The, 615
Durham County Nursing Association, 503
Dysenteries, The, 57

East Anglian Sanatorium, 586
**EDITOR'S LETTER-BOX, THE:**
"Are Many Hospitals to be Closed?" 331
Association of Hospital Matrons, The, 193, 227
Barrasford Sanatorium's Finances, 19
British Farmers' Hospital, The, 159
Church and the Marriage of Syphilitics, 26, 331
College of Nursing, The, 505
College Salaries' Committee, The, 45
Dogs' Protection Bill, The, May 3, vi
Factory Nurse, Position of the, 72
Fever Nursing, A Supplementary Register for, 138
Gout, Further Meditations on, 529
Gum-Saline in Wound-Shock, 138, 159
Hospital Reconstruction and Rebuilding, 529
Infectious Diseases, Fees for Notification, 19
Islington War Memorial, 331
Leeds Workpeople's Hospital Fund, 20
Norfolk and Norwich Hospital, 194
Nurses and Trade Unions, 275
Nursing Profession, The: Reconstruction *v.* Destruction, 19
Nursing Staff Salaries, 45
Patients' Livings, What Happens to, 355
Physiognomy, 395
Private Nurse, Working Hours of the, 159, 193, 252
Queen Charlotte's Hospital and its Nursing Staff, 619, 641
Ranyard Nurses, 642
St. George's Hospital, 506
Sister-Tutor, The Duties of a, 417
Staff Conditions in Hospitals, 642
State Registration, 45, 46, 160, 331, 505
Syphilitics, The Marriage of, 395, 417, 437
V.A.D.s at Casualty Clearing Stations, 227; Victimisation of, 619

Editor's Letter-box.—*continued.*
Victory Parade and After, The, 461
Who Can the Lady Be? 355
Women's Holidays, 437
Edmonton Infirmary, 503
Education, The Magic of, 393; In Hospital, 424
Educational Number of The Hospital, 551
Egg Production in a Fever Sanatorium, Supp. May 17
Egypt, The Native Hospitals in, 77; Ophthalmic Education in, 555
Eight-Hour Day for Nurses, 157, 329, Supp. June 28
Electro-Therapeutic Rooms, Equipment for, Supp. September 27
Embroidery for Patients, 158
Escape, The, 485
Essex County Nursing Association, 329
Eugenic Problem, A, 82
Examinations, The Plague of, 431
Exeter Cathedral, 458
Exeter District Nursing Association, 92
Experiments on Animals (*see* Vivisection)

Family Endowment Commission, 492
Fatigue, The Study of, 537
Fazakerley Fever Hospital, May 10, viii
Fees, Doctors' Increased, 531
Fellowship of Medicine, 583
Felony, The Interpretation of, 237
Film (*see* Cinema)
Filter-passing Virus, The, 582
Finch, Dr. E. Fen, 124
Finsen Light, A Substitute for, 245
Fires: Leeds General Infirmary, 6; Risks of Decorations, 403
Fitzgerald, Miss Alice, 226
Fleas, Prevalence of, 489
FOOD: Report of Royal Society Committee, 68; Rationing Register, 73; Prices, 158, 416; Organisation of Rationing, 273; Improvement of Supplies, 314; Accessory Factors, 371; The Preserving of, 473; Prospects, 548
Forthcoming Events: May 10, viii; August 23, iv; 622
Fourth Southern General Hospital, 113
Fractures, The Care of in Civil Hospitals, 339
Franklin, Mr. G. C., 259
Franklin, Mr. Philip, 143
Fry, Mr. E. C., 4
Funding Loan, 295

Garden-party at Buckingham Palace, 457
General Medical Council, 237
General's Criticism, A, 610
German Nurses, 503
Germany, Food Conditions in, 635
Gifts and Bequests, 93, 109, 221, 395, 418, 461, 484, 506, 550, 622, 635
Gillingham Sister, 192
Gipsies, Confinements amongst, 492
Girton College, 459
Glasgow Medical Schools, 514
Gledhow Hall V.A.D. Hospital, 169
Gloucestershire County Council, 4
Goldsmiths Company, 235
Goschen, Viscount, 131
Gout, A Modern Idea of, 104
Graduate Medical Instruction, 553, 554, 590
Green, Dr. Albert, 123
Green, Sir Frederick, K.B.E., 401
Greenwich Union Infirmary, 536
Guilds of Service, 440
Gunshot Fractures of the Femur, 347

Haddington V.A.D. Hospital, 329
Hadley Hospital, 373
Haig, Sir Douglas, 339
Hammersmith Borough Council, 597
Hampshire, Mr. C. H., 470
Harkness, Dr. R. C., 53
Harvey, Mrs. Martin, 91
Hawthorne, Dr. C. O., 280, 338
Health and the Army, 625
Health, Ministry of (*see* Ministry)
Health Visitors, 434, 586
Heatstroke and Allied Conditions, 631
Hertford and Ware Joint Hospital Board, 640
Hertfordshire County Council, 170
Hertfordshire Sanitary Report, 489
Hilling, Sister Sophie, 618
Hincks, Sarah, 18
Hocking, F. A., 31
Holidays, After the, 489; Nurses', 527; Holiday Funds, 535
Holywell Board of Guardians, 157
Home Helps, Training of, Supp. June 14, July 5, July 26
Home which the Builders Rejected, The, 625
**HONOURS:** The Mania for Orders, 78
Albert Medal, 413
Birthday, 513
British Empire, 434
Croix de Guerre, 483
Order of St. John, 67
Mentioned for Valuable Services, 91, 526, 545
Military Medal, 458
New Year Honours, 99
1914-1915 Star, 640
Royal Red Cross, 15, 41, 66, 67, 132, 189, 190, 192, 223, 247, 248, 272, 294, 352, 413, 458, 480, 501
War Medal, 431, 480, 506, 525
Hospital Airships, 408
**HOSPITAL AND INSTITUTIONAL NEWS**, 3, 29, 53, 77, 99, 121, 143, 155, 156, 167, 201, 235, 259, 281, 315, 339, 371, 401, 421, 445, 467, 489, 513, 533, 561, 583, 603, 625
Hospital and Institutional Results: 232, 462
**HOSPITAL ARCHITECTURE AND CONSTRUCTION:** (*see also* "New Era, The"); Stockport Infirmary, 245
Hospitals and their Special Needs, 309
**HOSPITALS, INSTITUTIONS, ETC.:** (Poor Law, War, Temporary and Auxiliary Hospitals. *See under* Individual Names)
Metropolitan:
Alexandra Hospital, 194
American Hospital, 143, 401, 435
Anti-Vivisection Hospital, 492
Belgrave Hospital for Children, 300
Bethlem Hospital, 31
Bolingbroke Hospital, 170, 194
British Home for Incurables, 158
Cancer Hospital, 73
Charing Cross Hospital, 22
Chelsea Hospital for Women, 118, 424, 442
City of London Hospital for Diseases of the Chest, 118
City of London Truss Society, 101
East London Hospital for Children, 163
Elizabeth Garrett Anderson Hospital, 274, 284
Florence Nightingale Hospital, 462
Freemasons' Hospital, 55, 284
General Lying-in Hospital, 259
Gloucester Royal Infirmary, 424
Great Northern Central Hospital, 56, 202, 204, 402, 421, 621
Grosvenor Hospital for Women, 194
Guy's Hospital, 96, 167, 232, 282, 315, 320, 403, 458, 483, 609
Hampstead General Hospital, 194, 421, 462
Hornsey Cottage Hospital, 342
Hospital for Consumption, Brompton, 44, 48, 191, 638, 640
Hospital for Diseases of the Heart, 163

Hospitals, Etc.—*continued.*
Hospital for Sick Children, 123, 273, 300
Hospital for Throat Diseases, 73
Hospital for Tropical Diseases (*see* Seamen's Hospital Society)
Hospital for Women, 48
Hostel of God, 462
Infants' Hospital, 73
Italian Hospital, 48
Jewish Maternity Home, 226
King's College Hospital, 16, 481
London Homœopathic Hospital, 73, 225
London Hospital, 16, 21, 43, 66, 100, 235, 252, 424, 462, 599, 610
London Lock Hospital, 48
London Temperance Hospital, 48
Metropolitan Hospital, 32
Middlesex Hospital, 3, 42, 48, 167, 189, 503
Miller General Hospital, 356
Mount Vernon Hospital for Consumption, 22
National Hospital for the Paralysed and Epileptic, 284
National Orthopædic Hospital, 48
Nelson Hospital, Wimbledon, 134
Paddington Green Children's Hospital, 284
Poplar Hospital for Accidents, 22, 54
Princess Club Hospital, 29
Queen Charlotte's Hospital, 16, 149, 250, 422, 469, 619, 620 641
Queen Mary's Hospital, 152, 434
Queen's Hospital for Children, 194
Royal Ear Hospital, 87
Royal Eye Hospital, 300
Royal Free Hospital, 22, 415
Royal Hospital for Diseases of the Chest, 462
Royal Hospital for Incurables, 145, 268, 442, 483
Royal Waterloo Hospital, 168, 194
St. Bartholomew's Hospital, 17, 189, 224, 316, 372, 610
St. George's Hospital, 31, 63, 133, 170, 189, 418, 462
St. John's Hospital, 194
St. Luke's Hospital, 203
St. Mary's Hospital, 82, 191, 225
St. Paul's Hospital, 73
St. Peter's Hospital, 73
St. Thomas's Hospital, 113, 155, 372, 391, 392, 428, 476, 483, 610
Seamen's Hospital Society, 4, 122, 146, 201, 237, 404
South London Hospital for Women, 170, 194, 249, 403
University College Hospital, 22, 87, 284, 610
Victoria Hospital for Children, 194, 300
Walthamstow Hospital, 423
Weir Hospital, Balham, 416
West-End Hospital for Nervous Diseases, 163
West London Hospital, 145, 168, 203
Westminster Hospital, 36, 37, 226, 328
Provincial, Scottish and Irish:
Aberdeen, Royal Hospital for Sick Children, 203, 639
Alton, Crippled Children's Hospital, 143, 250, 372
Ancoats Hospital, 442
Ashford Cottage Hospital, 6
Bangor Hospital, 260
Barnsley, Beckett Hospital, 249, 262
Bedford County Hospital, 597
Belfast, the Benn Ulster Eye, Ear and Throat Hospital, 102, 260; Royal Victoria Hospital, 113; Ophthalmic Hospital, 260
Birmingham, Queen's Hospital, 54; General Hospital, 67, 462; St. Chad's Hospital, 86; Skin Hospital, 145; Hospital for Nervous Diseases, 259
Blackpool, the Victoria Hospital, 79
Bognor, Cottage Hospital, 422
Bolton Infirmary, 4

HOSPITALS, ETC.—*continued.*
Boston Hospital, 55
Bournemouth, West Hants Hospital, 238, 341
Bradford Royal Infirmary, 134; St. Luke's Hospital, 318
Brighton, Sussex Hospital for Women and Children, 203; Royal Alexandra Hospital for Sick Children, 262
Bristol, Homœopathic Hospital, 260; General Hospital, 392
Canterbury, Kent and Canterbury Hospital, 65, 548
Cardiff, King Edward VII.'s Hospital, 55, 202, 281, 372, 403, 418, 440, 441, 469, 470, 515, 604, 618, 628
Chelmsford, Essex County Hospital, 469
Chester Royal Infirmary, 238
Chesterfield Royal Hospital, 123
Colchester, Essex County Hospital, 113, 169, 491
Coleraine Cottage Hospital, 462
Colwyn Bay Cottage Hospital, 404
Coventry and Warwickshire Hospital, 16
Croydon Hospital, 491
Dorchester County Hospital, 158
Dublin Hardwicke Hospital, 448, 549; Richmond Hospital, 448, 549; Whitworth Hospital, 448, 470, 549
Dumfries, Crichton Royal Institution, 462
Edinburgh, Royal Infirmary, 329
Enfield Hospital, 30, 238, 516, 628
Essex County Hospital, 462
Exeter, Royal Devon and Exeter Hospital, 121, 232, 274, 491
Glasgow Hospital for Disease of the Ear, Throat and Nose, 341; Royal Hospital for Sick Children, 354; Royal Infirmary, 442, 504; Ophthalmic Institution, 462
Gloucester, Royal Infirmary, 639
Gravesend Hospital, 113
Hartshill, Cripples' Aid Society Hospital, 492
Hastings, East Sussex Hospital, 404
Hereford Cottage Hospital, 56
High Wycombe Memorial Hospital, 73
Ipswich, East Suffolk Hospital, 4, 146, 404, 504
Kidderminster Infirmary, 5
King's Lynn, West Norfolk Hospital, 169
Kirbymoorside Cottage Hospital, 146
Leamington, Warneford Hospital, 318
Leeds General Infirmary, 4, 6, 80, 91, 202, 391, 392, 447
Leicester Royal Infirmary, 48, 416, 441, 462, 509, 600
Levenshulme, Babies' Hospital, 144
Lincoln County Hospital, 79, 226
Liverpool, Royal Infirmary, 91, 101, 102, 528; Heswall Children's Hospital, 421; David Lewis Hospital, 459; Infirmary for Children, 491; Country Hospital, 491; Samaritan Hospital for Women, 491
Loughborough Hospital, 585
Lowestoft Hospital, 123
Maidstone, West Kent Hospital, 628
Manchester, Babies' Hospital, 5
Mansfield and District Hospital, 32
Margam Cottage Hospital, 470
Margate, Royal Sea Bathing Hospital, 422
Melksham Cottage Hospital, 6
Merthyr General Hospital, 535
Monsall Hospital, 144
Newcastle-on-Tyne, Royal Victoria Infirmary, 78, 340, 618
Newport, Royal Gwent Hospital, 540
Norwich, Norfolk and Norwich Hospital, 123, 422
Nottingham General Hospital, 261
Oxford, Radcliffe Infirmary, 340
Peterborough Infirmary 124; War Memorial Hospital, 341
Plymouth, Ear and Throat Hospital, 491
Porth Cottage Hospital, 123

HOSPITALS, ETC.—*continued.*
Portsmouth, Royal Hospital, 113, 124, 134, 422, 605; Ear and Eye Hospital, 422
Preston Infirmary, 260
Purley Cottage Hospital, 170
Reading, Royal Berkshire Hospital, 123
Redditch, Smallwood Hospital, 32
Rhymney Cottage Hospital, 78
Rochester, St. Bartholomew's Hospital, 374
St. Leonards, Eversfield Chest Hospital, 462
Saltair Hospital, 4
Sheffield, Royal Hospital, 102, 328, 415
Shrewsbury, Salop Royal Infirmary, 6, 492
Southport, Moss Lane Hospital, 80; Homœopathic Cottage Hospital, 168
Stockport Infirmary, 6, 17, 245
Stoke-on-Trent, North Staffs Infirmary, 318, 492
Swansea Hospital, 123, 424, 516, 586
Torquay, Torbay Hospital, 5
Tunstall Cottage Hospital, 422
Ventnor, Hospital for Consumption, 48
Walsall General Hospital, 157, 516
Wellington Cottage Hospital, 55
Whitstable Cottage Hospital, 56
Wick, Bignold Cottage Hospital, 80
Wigan, Royal Albert Edward Infirmary, 43
Winchester, Royal Hants County Hospital, 17, 447
Woking, Victoria Hospital, 102
Wolverhampton and Staffs General Hospital, 4, 232
Worcester Infirmary, 448
Wrexham Orthopædic Hospital, 91
Yarmouth Hospital, 192
York County Hospital, 17, 329, 606
COLONIAL, U.S.A., &c.:
Grahamstown, South Africa, 490
Jerusalem, 286
Melbourne, Austin Hospital for Incurables, 548
Natal, Guy's Hospital, 462
Peshawar, C.M.S. Hospital, 401
Sydney, Prince Alfred Hospital, 404
Hospital News in Local Papers, 469
Hospital Saturday Fund, 100, 260
Hospital Sunday Fund, Special Number, June 21, 301, 416, 506
House of Lords Proves its Value, The, 200
**HOUSING**: 54; Bill, 149, 207; Some Present-Day Life Conditions, 484; Ministry of Health Action, 514; Inner London, 515; Nurses' Cottages, 546; The Crown and Lambeth, 583, 584; Tenement Houses, 594, 632; Nottingham, 627
Human Temperaments, 175
Hydrophobia, The Control of, 428

Imperial War Museum, 526
Income Tax Liabilities and Claims, 541
Incorporated Association of Hospital Officers, 31
Incorporated Society of Trained Masseuses, 528
India, Headquarters' Failure in, 531 (*see also* Afghan War)
Indian Hospital Insufficiencies (*see also* Afghan War), 445, 467
Individual and the Homestead, The, 257
Industrial Accidents, The Causes of, 222
Industrial Fatigue Board, 618
**INFANT WELFARE AND MATERNITY**: Red Cross Council, 18; Nurseries for Children, 68; National League, 68; Work in Southwark, Supp. April 19; Nottingham, 80; In America, 100; Hospital Beds for Babies, 144; Hertfordshire, 170; Deptford, 191; Camberwell, 260; National Birth-rate Commission, 391; National Baby Week, 394; Ante-Natal and Intra-Natal Care, 420; Queen's Visit to Centre, 414; The Super-Child, 455; Maternity Wards in Workhouses, 490; Confinements of Gipsies, 492; Portsmouth Municipal Maternity Hospital, 516; Edmonton, 533; Richard Murray Hospital, 535; Maternity Homes, 597; Pensions for Mothers, 621
Infectious Soldiers, Return of, 143
Influenza, 5, 53, 81, 344, 391, 459, 482, 533, 586, 603
In-Patient Case Records, Supp. April 12, 19, Supp. April 26
Insane (*see* Mental)
INSTITUTIONAL FACTS AND FIGURES (*see* The Institutional Worker—the Supplement to THE HOSPITAL)
INSTITUTIONAL LIBRARY (*see* Book World)
Institutional Treatment, Dangers of, 325
Insurance, Faculty of, 53
International Health Board, An, 29
Invalid Children's Aid Association, 203, 374
Ireland: Public Health In, 603; Nursing Affairs in, 614, 636
Irish Asylum Strike, The, 317
Irish Nursing Affairs, 21
Isleworth, Percy House Red Cross Hospital, 598
Ivory Cross National Dental Aid Fund, 203

Jaundice and its Cause, 343
Jerusalem, Hospital Life in, 286
Johnson, Dr., on Administration, 204
Jones, Mr. J., 491
Judge's Stricture on Doctors, A, 516

Kata-Thermometer, 264
Kealy, Miss E. M., 226
Keogh, Sir Alfred, 236
King Edward's Hospital Fund for London, 16, 39, 97, 99, 119, 135, 379
King Edward's Hospital Fund for the Midlands, 3
King George's Fund for Sailors, 167
KING, THE: 99, 341, 383, 457; Visit to Wales, 468; Water Pageant, 475
Kingswood and District Nursing Association, 392
Kipling, Mr. Rudyard, 477
Kipps, The Apotheosis of, 23
Knee, Surgery of the, 451
Know all the Facts, 490

Labour, International Adjustment of, 481
Labour Party and a State Medical Service, The, 98
Labrador Coast, Influenza on, 482
Lady Minto's Indian Nursing Association, 459, 527
Laing, Miss, 80
Lambeth Board of Guardians, 606
Lapthorn, Mr. T. H. F., 124
League of Mercy, The, 235, 401
League of Nations, Nurses and, 131
League of Red Cross Societies, 167
Leeds Poor-Law Infirmary, 640
Leftwich, Dr. Ralph W., 8
Legacies, The Cultivation of, 422
Legal Notes for Institutional Workers, 106
Lennox, Lady A. Gordon, 191
Leprosy in India, 152
Lest We Forget! 246
Libraries of Hospitals, 268; A Circulating Library, 446
Life of a Nurse, The, 47, 228
Limbless Soldiers, Hospitals for, 78
Linen Losses in Hospitals, 17
Lion Hospital Society, The, 56
Lister Institute, The, 253
Liverpool: Council of Voluntary Aid, 79; Joint Nurse Training, 224; University, 236; Health of School Children, 446

Livingstone College, 402
Local Anæsthesia, 446
Local Government Board, 176; (Scotland), 274
Lombardini, Rev. A., 95, 112, 132, 191
London and the Influenza Outbreaks, 587
London, Bishop of, 328
London County Council, 341; School Care Committees, Supp. July 12; Venereal Disease, 442; Canadian Soldiers, 481
London (Royal Free Hospital) School of Medicine for Women, 372
Lord Leverhulme's Hospital, 72
Louse Bites, The Effects of, 436
Luckes, Miss, 16, 462
Lumbar Puncture, The After-effects of, 497
Lunacy (*see* Mental)

McCarthy, Dame E. M., 353, 501
McDade, Dr. C. E., 259
Macdonald, Miss B., 330
Macmillan, Miss, 68
Madness (*see* Mental)
Malaria in England, 513, 602
Manchester Board of Guardians, 261
Manchester Exhibition and Conference, The, 333, 548, 617
Manor Mental Hospital, The, 605
Margarine, 206
Marr, Mr. James, 123
Marriage and Tuberculosis, 284
Marriage: Of Master and Matron, 31; Of Sanatoria Superintendents, 423
Masonic Hospital Service, 492
Massage and Masseuses: Army Masseuses, 44, May 24, v
Maternity (*see* Infant Welfare)
Matrons, Association of (*see* Association of Hospital Matrons)
**MATRONS' AND SISTERS' DEPARTMENT**, 15, 41, 65, 89, 111, 131, 155, 189, 223, 247, 271, 293, 327, 351, 389, 413, 431, 457, 479, 501, 525, 545, 595, 615, 637
Matrons, Well-paid, 413; Specially Trained, in Demand, 525; Worn-out, 547
Measles, Immunisation against, 240
Mechanical Genius and the Pen, The, 94
Medical Curriculum, The, 568
Medical Defence Union, 78, 626
Medical Education: Subsidies, 467; At London Hospitals, 609
Medical Missionary Exhibition, 341
Medical Outlook, The Certainties and Uncertainties of the, 551
Medical Parliamentary Committee, 130
Medical Parliamentary Union, 99
Medical Protection, 393
Medical Research and the Student, 557
Medical Research Committee, The, 9
Medical School: Difficulties in the, 316; A Floating, 340
Medical Student and his Work, The, 571
Medical Students, 416
Medicinal Men, The Future of, 282
Melbourne: Royal Victorian Trained Nurses' Association, 392; Nurse Training in, 503
Mental: Nursing, 47; Cinemas, 56; The Clothing of Lunatics, 106; Nursing Homes, 415; Madness and Marriage, 466; Uncertifiable Cases, 506; Increased Cost of Lunatics, 584, 606
Mercier, Dr. Chas. A., 20, 561, 591, 592, 593
Merthyr Guardians, 618
Mesopotamia, The Future of, 99; Nursing Services in, 156
Messum, Miss A. M., 65
Metropolitan Asylums Board, 77, 249, 502, 526, 596
Metropolitan Tuberculosis Difficulties, 120
Michelli, Mr. P., 122
Middle Class, Nursing the, 353
Midwives: St. Thomas's Hospital, 113; Still-born Children, 144; Claims Under Midwives Act, 447; Free Midwives, 550
Mile End Guardians, 637
Military Hospital, The Life of the Staff in a, Supp. May 3
Military Patients, Transference of, 441
Milk Supply, The, 483
Millicent Sutherland Ambulance, 597
Million for the Hospitals, A, 601
Ministers of Health, 500, 524, 544
Ministry of Health: Sir Robert Morant, 3; Position of Women, 133; The Ministry and the Medical Profession, 199; Voluntary Hospitals, 237; Hospital Sites, 267; Registration, 327, 339; National Baby Week, 394; Women in Ministry, 414; Health Visitors, 434; Consultative Councils, 439; Medical Appointments, 445; "Emergency" Health, 468; The Nursing Profession, 478; Nursing Service, 480; Regional Pensions System, 488; The Housing Question, 514; Assistant Inspectorships, 526; Local Staffs, 603; Venereal Disease, 604; Tuberculosis Officers, 627; Probationers' Salaries, 637; Nursing Salaries, 638
Ministry of Labour, 225
Ministry of Pensions, 53; Sir Douglas Haig, 339, 371; Ten-minute Cures, 402; Problems, 444; Hospital Accommodation, 445
Ministry of Reconstruction, 68, 565, 599
Ministry of Scientific and Industrial Research, May 24, v
Mistakes, Reports of, 202
Mitcham Ambulance, 73
Moore, Mr. Francis, 101
Morant, Sir Robert, 3
Morphia, British-made, May 24, v
Morris, Mr. E. W., 100
Morris, Sir Malcolm, 146
Motherhood, The Endowment of, 426
Mothers' Pensions, 67
Motor Ambulance Dinner, The, 201
Moynihan, Sir Berkeley, 638
Murder of a Nurse, 459, 503

Names, The Choice of, 2
National Association for the Prevention of Infant Mortality, 483, 597
National Baby Week (*see* Infant Welfare)
National Birth-rate Commission, The, 295
National Council of Women, 68, 133, 616
National Health Insurance: Bonus for Panel Doctors, May 31, iv; Bonus Increased, 331; Panel Doctors and the Hospitals, 456, 477; Dangers of Big Panels, 512; Limit, 524, 527; Changes, 540
National Institute for the Blind, 318
National League for Health, 68
National Relief Fund, 23
National Roll of the Great War, 596
Nation's Fund for Nurses (*see* College of Nursing)
Native Regiments, Hospitals in, 118
Navy, Notes of a Surgeon, 59, 151
Navy Nursing Sisters, Discharges, 156; Rates of Pay, 477, 596
Needles in the Heart, 489
Netley Hospitals, 101
Netley Red Cross Hospital Magazine, 80
Neurasthenia and its Cure, 543
Neurasthenic, The Case of the, 325
New End Military Hospital, 16
New Era, The, 61, 153, 269, 411, 523, 613
New Zealand Nursing Reform, 598
Newport Board of Guardians, 124
Newspapers as Germ Carriers, 73
Newton Abbot Board of Guardians, 121
Night Nurses' Meals, 439
Nightingale, Florence, 3, 134
Norfolk Nursing Federation, 225
Norfolk Sanatorium Benefit Committee, 62
Norman, Sir W. H., 167
North-Eastern Hospitals' Association, The, 56, 627
North Wales Nursing Association, 43
Northern Hospital, Winchmore Hill, 114
Norwich, Shop Collections in, 534
Novice in General Practice, The, 532
Noyes, Miss C. D., 640
"Nurse-Chauffeuse-Secretary," 330
Nurse Training Schools, 639
Nurses' Accommodation in Hospitals, May 10, viii
Nurses' Day, 90
Nurses Disabled in Former Wars, 397
Nurses' Insurance Society, 250
Nurses' Off-Duty Times, 43, 134
Nurses' Resettlement and Demobilisation Committee, 112
Nurses' Union, The, 92
Nursing and Midwifery Exhibition and Conference, 117, 133, 139
Nursing Homes, Mental Cases, 415
Nursing Service, Inadequacy of, 152

OBITUARY: Sir Wm. Crooks, 29; Dr. A. G. Bateman, Col. J. A. Jones, 92; Dr. E. F. M. Finch, 124; Mr. A. Watkins, May 10, viii; Sir E. Stirling, 143; Mr. S. Perkins Pick, 202; Dr. W. Cottle, 237; Mr. G. C. Franklin, 259; Colonel Bruce Vaughan, 315; Dr. F. Collins, 423; Mr. Joseph Summers, 492; Dr. Charles Mercier, 561; Sir P. W. Squire, 626
Ontario Military Hospital, Orpington, 317
Ontario, The Exceptional Child in, 510
Open-Air School Shortage, 144
Open Grate, The, 447
Operating-Theatres, The Heating of, 110; The White Theatre, 624
Ophthalmology, Teaching of, 236, 316
Opium, Indian, Use of in European Medicine, 467
Optimum Population, An, 176
Orde, Mr. Robert, 78
Order of the British Empire (*see* Honours)
Orders (*see* Honours)
Osler, Sir William, 339, 409, 515
Oversea Nurses, 93, 126, 355, 391

Paddington and St. Marylebone District Nursing Association, 159
Paddington Military Hospital, 92
Paget, Mr. Stephen, 503
**PARLIAMENT AND THE PROFESSION**, 23, 47, 64, 93, May 10, viii, 152, May 24, v, May 31, iv 267, 397, 418, 441, 477, 506, 540
Part-time Nurse, The, 354
**PASSING EVENTS AND TOPICS**, 73, 116, 198, 227, 442, 483, Aug. 30, viii, 621
Patent Medicines, Advertising, 201
Patients' Leavings, 261, 424
Peace: Its Coming and its Awakening Powers, 319; Peace and the Profession, 370; Celebrations, 340; Thanksgiving Service, 372, 383, 390; July 19th Celebration, 419, 433; Nurses in the Procession, 414, 433
Pearls in Food, 630
Pearson, Sir Arthur, 318
Pensions: King Edward's Hospital Fund Action, 16, 39
Pensions Problems, 444
People's League of Health, 143
Personal Service, The Power of, 119
Peterborough War Memorial Committee, 585
Peterborough, The Parable of, 79
Pharmaceutical Society of Great Britain, 583
Physical Education, 401
Physiognomy, On, 291
Pick, Mr. S. Perkins, 202

Picture Palaces, The Control of, 341
Pink, Sir Harold, 605
Pirie, Dr. G. H., 123
Plague (*see* Bubonic)
Plaster Work, 538, 608
Play, The Need for, 52
Plymouth: Three Towns Nursing Institution, 158
Pneumonia Mortality, The, 81
Pocket-handkerchief, The, 10
Poison in the Cup, The, 208
Poland: The Minister of Health, 513; Typhus Situation, 639
Policy of Waiting, The, 487
Poor Law (*see also* Local Government Board and Ministry of Health)
Poor-law Buildings and State Hospitals, 27
Poor-law Hospitals, Army and, 597
Poplar Board of Guardians, 190; Measures of Disinfection, 518
Population, The Decline in, 142
Port Restrictions, Proposed, 533
Ports and Disease, 54
Portsmouth Board of Guardians, 354; Municipal Maternity Hospital, 516
Postcard, The Unpardonable, 209
Post-graduate Teaching, 28, 100, 583
Poultry and German Prisoners, 124
Powell, Sir R. Douglas, 353
Presentations, 600, 627
Preventive Medicine, Teaching of, 148
Prince of Wales, 31, 54, 97, 99, 119, 167, 545
Princess Christian, 353
Princess Club Hospital, 29
Princess Mary, 133, 190
Princess Marie Louise, 29
Private Nursing, The Eight-Hour Day in, 637
Probationers: Age of Entry, 191; The New Spirit, 457; Physical Side of Training, 479; The Art of Attracting, 615
Professional Secrecy, 258, 280
Prohibition or Freedom, 399
Propaganda, 412
Public Health, Reconstruction in Relation to, 245; Lessons of the War, 263; Services, 564; London in 1918, 626
Public School Hospital, 3
Pugh, Mr. Evan, 78

Queen, The, 16, 134, 190, 272, 341, 383, 414, 457, 475
Queen Alexandra, 16, 66, 134, 157, 237, 414, 458, 480
Queen Alexandra's Imperial Nursing Service: New Rates of Pay, 156, 191, 562; Work in India, 415, 460
Queen Alexandra's Royal Naval Nursing Service, 224, 562, 596
Queen Mary's Auxiliary Army Corps, 415
Queen Victoria Jubilee Institute for Nurses, 43, 157, 415, 459
Quien, Trial of, 598

Rabies, 77, 103, May 24, v, May 31, iv
Radiography, War, 427
"Ragging" at an Infirmary, 192, 226, 391
Ranyard Nurses, 598
Rats as Carriers, 514
Recent Official Publications, 46; April 26, vi, May 3, vi, Aug. 16, viii
Recruits, Physical Condition of, 477
Red Cross, Gift to Gloucestershire, 4; Inter-Allied Conference, 44; And the Nurse, 64; Fur Trade Appeal, 73; The Doings of the, 109; Scottish Branch, 134; V.A.D. Scholarship Scheme, 248; Ambulance Home Service Scheme, 371; In Ireland, 430; Record of Work in Middlesex Hospitals, 470; Supplemental Charter, 480; Memorial to Motor Transport Men, 483; Money Gifts to Hospitals, etc., 601, 616
Rees, Dr. F., 122
Registration (*see* State Registration and College of Nursing)
Religion, The Body and Health, 605
Resident Medical Officers, Hospital, 515
Rickets as a Dietetic Disease, 7
River Past and God Forgotten, 92, 160
"River's Tale, The," 477
Rockefeller Foundation, 126
Roehampton Home Hospital, 414
Roll Call of the Sick, The, 302
Rooks in Brighton, 94
Ross, Miss E., 294
**ROUND THE HOSPITALS**, 15, 41, 65, 90, 112, 131, 155, 189, 223, 247, 272, 294, 327, 351, 389, 413, 431, 457, 479, 501, 525, 545, 595, 615, 637
Royal Academy, The, 294
Royal Air Force: Medical Officers, 468
Royal Army Medical Corps Memorial, 141; New Directorate, 121; Memorial Service, 167, 201; Demobilisation of Temporary Officers, 488, 559
Royal British Nurses' Association, 89, 368, 390
Royal Club of Women, 617
Royal College of St. Catherine, 481
Royal College of Science, 236
Royal College of Surgeons, 442
Royal Institute of Public Health, 283, 442
Royal National Pension Fund for Nurses, 328, 480
Royal Navy, The, 287; Medical Service, 558
Royal Sanitary Institute, 617
Royal Society of Medicine, 29
Russell, Rev. C. A. W., 121
Russia: Hospital Ships, 502, 506
Russian Red Cross, 353, 390, 416
Rutter, Mr. W., 102

St. Alban's Infirmary, 354
St. Dunstan's 407
St. Marylebone Infirmary, 329
St. Pancras Board of Guardians, 536
St. Pancras Infirmary, 16
Salaries, Sisters' and Nurses' (*see also* College of Nursing): Kidderminster Infirmary, 5; King's College Hospital, 16; London Hospital, 21; Middlesex Hospital, 42; Example of Inadequacy, 67; St. George's Hospital, 133; Bradford Royal Infirmary, 134; Walsall General Hospital, 157; London Homœopathic Hospital, 225; Beckett Hospital, 249; Westminster Hospital, Sheffield Royal Infirmary, 329, 415; Royal Free Hospital, 415; Guy's Hospital, 458; Mile End Guardians, 637; Ministry of Health, 638
Salvation Army, 606
Samaritan, The Bad, 79
Sambon, Dr. Louis, 340
Sanatoria (*see* Tuberculosis)
Sanitary Administrative Matters, Two, 31
School Nurses, 296
Schools, Medical Inspection of, 201
Scottish Women's Hospitals, 250
Self-determination for Nurses, 270
Session, The New, 625
Shadwell Park, 535
Sheffield, Joint Appeal for Hospitals, 102, 260
Sheppey Board of Guardians, 261
Sibthorpe, Mr. C. C., 79
Sick Children's Nurses, 273
Sitting Tight While Others Fight, 154
Skinner, Miss Frances C., 352
Slesinger, E. G., 83
Slums, The Clearing of the, 242; An Ingenious Celebration, 490
Smaller Hospitals, The Opportunity of the, 501
Smallpox, Our Defences against, 14; Differential Diagnosis, 150; In Germany, May 24, v; Glasgow, 283, 540
Smith, Professor G. E., 235
Smoking Habit, The, 172
Social Work, The Call of, Supp. July 19, 1919
Society for the Propagation of the Gospel in Foreign Parts, 460
Society of Chemical Industry, 437
Soldiers, Consumptive, 62
South Africa, Influenza in, 5; Tuberculous Soldiers, 5; War Memorials, 547
South African Hospital, 54
Southport: Death of Babies in a Nursing Home, 640
Southwark Board of Guardians, 262, 423, 446, 585
Southwark Borough Council, 46
Southwark Day and Night Nursery, 249
Special Police, Hospital Staffs and, 606
Spirit Duty, Hospitals and, 169, 176
Spirits, Certified Medical Supplies, 267
Squire, Sir P. W., 626
Staffing a Hospital of Fifteen Beds, Supp. Aug. 9, 16, 23, 30
Staff Surgeon, The, 169
State Controlled and Voluntary Hospitals, 122, 234, 235
State Medical Service, A: The Labour Party, 98, 418
State Registration of Nurses (*see also* College of Nursing), 1, 15, 24, 42, 49, 65, 67, 69, 88, 89, 111, 112, 131, 154, 200, 211, 247, 251, 275, 293, 294, 327, 328, 351, 361, 389, 396
Stephens, Canon J. O., 585
Steward, Passing of the, 6
Still-born Children, 144
Still, Miss Lloyd, 155, 161
Stirling, Sir E. C., 143
Stockport Schools and the Military Octopus, 585
Stoke Poor-Law Hospital, 460
Street Collections on the Wane, 536
Strikes of Nurses, 113
Strong, Dr. H. J., 423
Suicide and Imitation, 514
Summer Fetes, The Organisation of, 422
Summer Time, 233
Summers, Mr. Joseph, 492
Super-Child, The, 455
Suppuration, The Treatment of, 83
Sussex County Nursing Association, 392
Swansea Nursing Association, 18
Syphilis (*see* Venereal Diseases)

Tenement Houses, 594, 632
Ten-minute Cures, 402
Territorial Nursing Service, 133
Tetanus and British Troops, 93
Thain, Sir George, 235
Thermometers, Inaccurate Clinical, 23
*The Times* and Medical Advertisements, 282
Thies, Mr. Conrad, 20
Third London General Hospital, 121, 447
Thomas, Sir J. L., 274
Thyroid, Minor Insufficiency, 125
Tipton and Ocker Hill Nursing Association, 416
Toogood, W. F. S., 424
Toone, Mr. F. N., 585
Town Life, Some Penalties of, 498
Training-school for Nurses: The Modern London, 189
Training-schools and Business Habits, 41
Tubercle, The 62
**TUBERCULOSIS**: Red Cross Gift to Gloucestershire, 4; South Africa, 5; Industrial, 31; Tuberculosis Topics, 62, 222, 350; Cannes Conference, 77; Subsidies, 84; Metropolitan Difficulties, 120; Special

Journal for Nurses, 122; Colliers' Immunity from Phthisis, 129; National Insurance Patients, 226; Observation Beds for, 238; Substitute for Finsen Light, 245; Soldiers and Sailors, 259; Marriage, 284; Airship Cure, 296; New Sanatoriums in Cheshire and Lancashire, 342; The War, 378; Early Visitation, 402; Sanatorium for Nurses, 459; Compulsory Segregation, 474; New Essex Sanatorium, 483; Soldiers on the Land, 521; Local Decline, 534; Care Committees, 534; Homes for Chronic, 534; Private Practice, 542; Open Windows, 548; Conference, 603; At Home and Abroad, 623; Ministry of Health, 627
Turner, Mr. William, 146
Twilight of the Gods, The, 456
Twilight Sleep, 29
Tynemouth Poor-Law Hospital, 528

Under-Five Citizen, The, 115
Uniform System of Accounts, The, 20
Uniforms, Protected Nurses', 458
United States: Red Cross Nurses and the Influenza, 73; American Optimism, 126; Commemoration Day, 190, 226; Graves of Nursing Sisters, 250; The Drug Habit, 283; Physical Education, 401; Army Rank for Nurses, 416; American Surgeons in France, 423; Red Cross Memorial Day, 460; Red Cross Demobilisation, 546; Health Ministry, 603; Multiplication of Hospitals, 626; Lectures, 640
University College, 235
University Training and Nurses, 351
Unqualified Nurses, The Future of, 545
Urban District Councils, Annual Conference of, 482

Vaccination: The Future of Therapy, 28; Value of, 168; Measures, 267; Fees for Public Vaccinators, 583
V.A.D.: Hospitals, 64; Gratuity to Nurses, 64; Demobilisation of Nurses, 69; Army Council, 112; Medal, May 24, v.; Red Cross Scholarship Scheme, 248; Welsh National Register, 274; National Health Act, 456; Alleged Victimisation, 535; Social Work, Supp. Sept. 13; Naval Service, 617; Reception at Harewood House, 638
Vaughan, Col. E. M. Bruce, 281
Vegetable Marrow, 528
Venereal Diseases, 23, 30, 208, 267, 313; Practitioners and the Marriage of Syphilitics, 338; Death under Treatment, 418; Early Preventive Treatment, 442, 468; Ministry of Health, 604; Need for Technical Opinion, 604
Victory Loan, The, 295, 353
Victory, The Two Conceptions of, 78
Vincent's Angina, 607
Vitamines, 285, 629
Vivisection, 53
Voluntary Hospitals' Budget, The, 302
Voluntary Hospital Essential to Public Safety, 589, 611, 633
Voluntary System, The Future of the, 337
Vote of Thanks to Army and Navy, 502

W.A.A.C., Hospital for, 440
Wales, The King's Visit to, 468
Wall Surfaces, 61
War and Surgical Progress, 171, 241, 319, 404, 495
War Bonus, The Elusive, 586
War Charities: Scotland, 23; Demobilisation, 30
War Experience and Civil Practice, 556
War Gratuities for Regular Officers, 10
Warlingham Military Hospital, 621
War Medals (*see* Honours)
War Memorials: R.A.M.C., 143; Lowestoft, 122; Berkshire, 123; Peterborough, 124; Bishop's Stortford, 168; Hythe, 168; Egham, 204; For Small Hospitals, 590; How to Pay for, 621
War Office and Doctor, 339
War Savings, 490
Wasp Stings (*see under* Bee)
Water Pageant, The Great, 475
Water Supply: On Active Service, 35; of London, 372
Watford Guardians, 598
Watkins, Mr. Arthur, May 10, viii
Waxol, 118
Webb, Sir Henry, 91
Welfare Teaching, Reform In, 406
Welfare Work in Factories, 638
Wellcome Historical Medical Museum, 483
Welsh Board of Health, A, 341
Welsh Medical School, 281
Welsh National Committee of Nursing, 638
Welsh National Eisteddfod, 80
Welsh National Memorial, 549
West Riding Nursing Association, 274
Whitechapel Board of Guardians, 536
Whitefriars Club, 133
Windsor Isolation Hospital, 114
Winsley Sanatorium, 62
Wittring Court Nursing Home, 296
Wolverhampton, Queen Victoria's Institute, 274
Women Doctors and the Winter Session, 626
Women in Industry, 499
"Women Who Win," 134
Women's World, 438
Worst War of the World, The, 287, 321
Wound Shock, 77, 85, 443

*X*-Rays and Cancer, 127

Young Men's Christian Association, 14, 324

PRINTED BY

SPOTTISWOODE, BALLANTYNE AND CO. LTD.

LONDON, COLCHESTER AND ETON.

### Dr. Ralph W. Leftwich.

We regret to announce the death, on March 25, after a very short illness, of Dr. Ralph Winnington Leftwich, of 36 Ebury Street, S.W. Dr. Leftwich was the author of a number of well-known medical books of real value to the student and general practitioner, not only from the knowledge they revealed, but also from their originality of treatment and the powers of deduction shown. Dr. Leftwich was also a keen student of Shakespeareana, and to his paper showing that St. Saviour's, Southwark, was Shakespeare's parish church and presumably his place of worship is due the erection of the Shakespeare Memorial there.

### THE COLLEGE OF NURSING. (Limited by Guarantee.)

### THE LEEDS CENTRE.

(Hon. Secretary, Miss Lindall, Women and Children's Hospital.)

A pleasant and successful "At Home" was given to the members of the Centre by the Committee on Thursday, March 20, at Collinson's Café, Albion Street, Leeds, when tea was provided. This, the first social function, concluded the pioneer year of the Centre. Miss Innes, R.R.C., Chairman and Local Representative, received the guests, assisted by the Committee, and gave a short address. It was a representative meeting, members coming from Hull, Harrogate, Huddersfield, Wakefield, York, Bradford, Shipley, and the outlying districts of Leeds. On admission each member was given a voting paper dealing with the meetings for next year, to ascertain the preference for lectures or discussions on strictly professional subjects; literary, historical, or other subjects, or purely amusements, such as sports, whist drives, dances, or picnics. Members were asked to send suggestions to the Hon. Secretary, Miss Lindall, who, in the course of her address, invited all the Members of the Centre to a whist drive at the Hospital for Women and Children, Leeds, on Thursday, April 24, from 7 to 10.30 p.m. All intending to accept are asked to send in their names a few days before the date to facilitate the making of the necessary arrangements. Each member is also asked to forward her annual subscription of 2s. 6d. to the Hon. Treasurer, Miss Edwards, Maternity Hospital, Leeds, on March 31 of each year, so as to save time and expense. This year each member is to have a membership card, which will be given as a receipt for the subscription, and which will admit her not only to her own Centre, its functions and lectures, but to any other Centre without extra fee. If lost, a new card will be obtainable only by paying a second subscription fee.

Fifteen of the members of the General Committee were re-elected. Six nurses (sisters) were elected on the new Committee, so now the nurses will feel that their colleagues are assisting in the organisation of the Centre. The officers were all re-elected: Miss Innes, R.R.C., Chairman and Local Representative; Miss Edwards, Hon. Treasurer; Miss Lindall, Hon. Secretary. Miss Spann, joint Hon. Secretary with Miss Lindall, resigned before the election owing to pressure of work, but she is still on the General Committee and the Representative for Poor-Law nurses.

For some time the Committee has recognised the desirability that so large a Centre as Yorkshire should have a nurse-representative on the Council in London. Last year seven or more candidates were nominated. At the meeting on the 20th ultimo the members, by means of votes, nominated a single candidate, for whom it is hoped they will vote in April.

### GLASGOW CENTRE.

Captain Alexander MacLennan, who lectured to members of the Centre and their friends on "War Surgery" at the Christian Institute, Bothwell Street, on March 18, illustrated his lecture by a series of most interesting slides. These showed how much can be done by surgery to repair the injuries done to our soldiers during the war.

The next meeting of the Centre will take place on April 15, at 8 p.m., at the same Institute, Bothwell Street, when Major Riddell, 4th Scottish General Hospital, Stobhill, will give a lecture on "*x*-ray Work." All nurses are cordially invited to be present.

### MANCHESTER CENTRE.

(Hon. Sec., Miss Earl, Ancoats Hospital.)

At a well-attended meeting held at the Royal Infirmary on March 28, Miss Sparshott, R.R.C., president of the East Lancashire Branch of the College, gave an interesting address on the College and its work, and was accorded a hearty vote of thanks. A social evening followed.

### CAMBRIDGE CENTRE.

(Hon. Sec.: Miss K. Newman, Addenbrooke's Hospital.)

On March 31 Captain McLachlan gave a lecture on venereal diseases in the 1st Eastern General Hospital, Cambridge. A large number of nurses were present. Sister Kate Taylor was in the chair. The next lecture, on April 25, also in the 1st Eastern General Hospital, will be on "The Treatment of Venereal diseases."

[We regret that owing to pressure on our space "The Institutional Worker" and other features are held over this week.]

